# Simple and universal function of acetic acid to overcome the drought crisis

**DOI:** 10.1007/s44154-023-00094-1

**Published:** 2023-05-26

**Authors:** Toru Kudo, Taiko Kim To, Jong-Myong Kim

**Affiliations:** 1 Ac-Planta Inc, Tokyo, Japan; 2 Department of Biological Sciences, The University of Tokyo, Tokyo, Japan; 3 Graduate School of Agricultural and Life Sciences, The University of Tokyo, Tokyo, Japan

**Keywords:** Acetic acid, Drought, Epigenetics, Jasmonic acid, Metabolism, Tolerance, Water deficit

## Abstract

Acetic acid is a simple and universal compound found in various organisms. Recently, acetic acid was found to play an essential role in conferring tolerance to water deficit stress in plants. This novel mechanism of drought stress tolerance mediated by acetic acid via networks involving phytohormones, genes, and chromatin regulation has great potential for solving the global food crisis and preventing desertification caused by global warming. We highlight the functions of acetic acid in conferring tolerance to water deficit stress.

## Introduction

Acetic acid is a carboxylic acid that is generally found as a metabolite in organisms. For a long time, human beings have produced and utilised this acid as vinegar. In addition to being used for food purposes, vinegar has also been used as an agricultural material, such as an herbicide, because of its acidity derived from acetic acid (Radhakrishnan et al. 2003; Johnson et al. 2003; Curran et al. 2004; Chandran. 2003; Chandran et al. 2004; Ivany 2010).

In plants, acetate is synthesised by the oxidation of acetaldehyde catalysed by aldehyde dehydrogenases (ALDHs) and then activated to acetyl-CoA by acetate-activation enzymes, including acetyl-CoA synthetase (ACS) and acetate non-utilising 1 (ACN1). A genetic study on ACS and ACN1 demonstrated that acetate homeostasis is maintained through acetate activation and it is crucial for plant development under normal conditions (Fu et al. 2020). Acetyl-CoA is an important acetyl donor for many biological reactions, including primary and secondary metabolism and protein acetylation (Tanner et al. 2000; Langer et al. 2002; Katada et al. 2012; Pietrocola et al. 2015) Since histone acetylation affects chromatin structure and transcriptional activity, acetate metabolism plays a pivotal role in the regulation of gene expression.

The physiological roles of the acetate-synthetic reaction, detoxification of acetaldehyde, and regeneration of nicotinamide adenine dinucleotide (NAD +) required for glycolysis under anaerobic conditions have been proposed (Kürsteiner et al. 2003). While Arabidopsis harbours 16 genes encoding ALDHs, ALDH2B4 would correspond to these functions in the hypoxic stress response (Fu et al. 2020). In addition to hypoxic stress, drought stress also induces acetate accumulation in plants (Kim et al. 2017). In the current model, increased acetate contributes to early adaptation to water shortage through histone acetylation and jasmonic acid (JA) signalling, as described in the following sections. Moreover, exogenous acetate enhances drought tolerance in a wide range of plant species, indicating its suitability as an agricultural material for controlling the risk of drought.

## Novel mechanism of plant drought tolerance by acetic acid

In plants, acetic acid, a fundamental metabolite in organisms, functions as a stimulator of drought tolerance and is correlated with gene activation and epigenetic regulation (Kim et al. 2017). Under water deficit conditions, the central metabolic flow from glycolysis to the TCA cycle is dynamically downregulated by gene expression levels and converted to acetic acid biosynthesis in *Arabidopsis thaliana*. Pyruvate decarboxylase 1 (*PDC1*) gene, which encodes pyruvate decarboxylase and aldehyde dehydrogenase (*ALDH2B7*) gene, which encodes aldehyde dehydrogenase, have essential functions in drought resistance via acetic acid synthesis from pyruvate. The conditional overexpression of these genes in plants shows resistance to water stress (Rasheed et al. 2018). Acetic acid stimulates temporal JA biosynthesis and promotes a JA-responsive gene network involving stress-responsive genes (Kim et al. 2017). Histone deacetylase (HDA6) known as an epigenetic regulator, directly regulates *PDC1* and *ALDH2B7* gene expression under water deficit conditions. On analysing the acetic acid–JA drought tolerant mechanism, abscisic acid (ABA), well known as a drought responsive phytohormone, was observed to not accumulate and gene expression of ABA responsive genes was not activated in *A. thaliana*. In the *hda6* mutant, higher accumulation of endogenous acetic acid by dry treatments was detected compared to that in wild-type plants, and the expression of ABA-responsive genes was notably delayed in the mutants.

This response via acetic acid against water deficit conditions is distinguished from well-known plant responses such as ABA-dependent and ABA non-dependent gene regulation networks, and the accumulation of osmolytes, including proline (Kishor et al. 1995). The genes for acetic acid biosynthesis and water deficit stress-response genes were activated by *hda6* mutation and exogenous application of acetic acid with water depletion in *A. thaliana* (Kim et al. 2017). On the other hand, the activation of dehydration-responsive element binding protein 2 (*DREB2A*), response to desiccation 29 A (*RD29A*)*,* and response to desiccation 29 B (*RD29B*) genes and proline accumulation were delayed responses of the acetic acid–JA drought tolerance mechanism under these conditions. Acetic acid promotes JA biosynthesis. However, the disruption caused by JA synthesis and JA regulatory gene networks results in the loss of drought tolerance in Arabidopsis. However, exogenous JA and methyl-JA applications were not effective in conferring plant drought tolerance (data not shown). Therefore, it appears that the effect of endogenous JA biosynthesis by acetic acid is different from that of conventional JA-induced injury responses in plants.

An important factor that can help understand this novel drought resistance system, is its relationship with stomatal opening and closing. Although a reduction in transpiration was observed; it did not result in growth enhancement or have any effect on deficit irrigation in maize (Allen and Allen 2020). In addition, there were no differences in stomatal opening and closing between the treated and untreated *A. thaliana* (unpublished data).

## The essence of epigenetic regulations mediated by acetic acid

To fully understand this novel mechanism, another important aspect, “epigenetic regulation,” is required. Histone acetylation is an activation marker of histone modifications that regulate chromatin structure and gene activity in eukaryotes (Fischle et al. 2003; Fukuda et al. 2006; Millar and Grunstein 2006; Kim et al. 2008). In particular, histone acetylation is correlated with the rapid ON and OFF regulation of drought-responsive genes in *A. thaliana* (Kim et al. 2008; Kim et al. 2011). The removal of acetylation at Lys 9 in the histone H4 N-terminus region is dynamically correlated with the RNA polymerase binding features in the transcribing regions. The application of exogenous acetic acid to plants before starting to stop water supplies as a water deficit treatment increases the genome-wide basal level of histone acetylation and is directly incorporated into histone H4 acetylation in *A. thaliana* (Kim et al. 2017). This report shows that histone acetylation occurs independently before gene expression following water deficit treatment. ACS mainly converts acetic acid to acetyl-CoA (Katada et al. 2012). The levels of acetyl-CoA generated using ACS correspond to the corresponding HAT enzyme activity (Tanner et al. 2000; Pietrocola et al. 2015). The high affinity binding of acetyl-CoA is a critical first step in histone acetylation by general control non-depressible 5 (GCN5). (Langer et al. 2002). Upon application of acetic acid to *A. thaliana*, ACS is highly induced by water deficit treatment (supplementary data in Kim et al. 2017). Therefore, the enrichment of acetyl-CoA occurs as a substrate for histone acetylation following acetic acid treatment in plants. The enriched regions of histone acetylation are pre-activated (priming) before becoming drought stress signals and rapidly and strongly respond to the addition of drought stress signals.

Thus, acetic acid has two functions: (1) as a stimulator of JA synthesis and (2) as an epigenetic priming factor for gene activation. Therefore, it seems that acetic acid application can create the “ready to go status” for gene activation on the genome-wide scale, prior to the stress condition being applied, and easily enhances the transcription of its target genes. As the potassium acetate produces the same effect as that of acetic acid and the pH of the xylem supernatant is in the neutral range, they were both applied (Ogawa et al. 2021). Acetic acid is used inside the plant body to act as an acetate ion.

## The new model of the process that responds to water deficit in plants

**Fig. 1:**
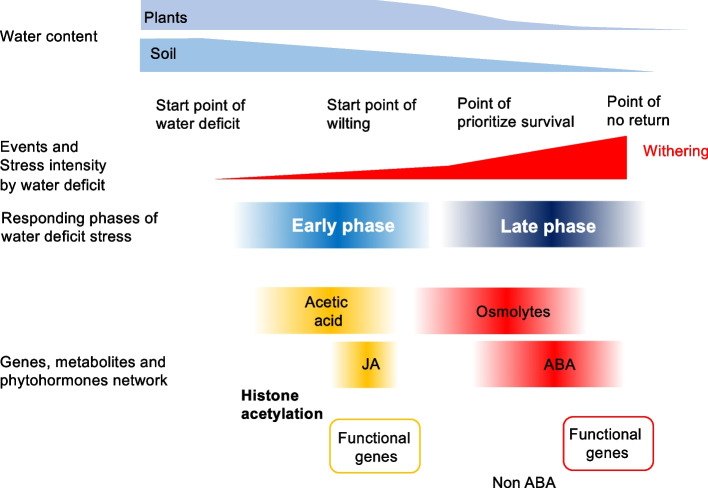
Schematic representation of response process to water deficit in Arabidopsis. At the early phase of response (under the moderate water-stress condition), when the water depletion from soil begins, but still adequate water remains inside of the plant’s body, chromatin regulation factor HDA6 functions like a switch molecule of gene expressions encoding these enzymes of acetic acid biosynthesis. Biosynthesis of acetic acid from pyruvate is promoted, enzymatically and dynamically converts the metabolic flow form glycolysis-TCA cycle to glycolysis-acetic acid biosynthesis. Acetate stimulates JA biosynthesis and JA responds to the gene network. At the same time, acetic acid is incorporated into the chromatin through histone acetylation to prime and activate the functional genes epigenetically. In the late phase of response (under hard water-stress condition), because of the progression of water depletion caused by the continuously prevailing strong dryness, heat, and salinity stresses, plants are unable to retain water inside their bodies. Osmolyte accumulation and ABA-dependent and -independent gene networks are promoted. When these late responses are activated, plants prevent the dry-up via decreased fluid flow and stomata closure. JA: jasmonic acid, ABA: abscisic acid

Based on the relevant results and our knowledge, we propose a new model for drought response in plants (Fig. 1). The plant drought response mechanism consists of early and late phases. The early phase refers to the gradual decrease in the water content of the soil; however, the plant body is still able to maintain sufficient moisture. The acetic acid–JA drought tolerance mechanism functions to maintain the water content inside the plant’s body during this phase. Thus, the early phase is the initial step for plant survival under moderate stress conditions.

The late phase refers to a significant decrease in the water content levels inside both the soil and plant body. ABA-dependent and ABA-independent gene regulation networks, as well as osmolytes, prevent physical damage to organs and even death, which can be caused by significant water loss inside the plant’s body under very harsh drought conditions. This model depends on the balance of water content within the plant body and soil.

It has been reported that the high expression of eukaryotic translation termination factor 1 (eRF1) can confer water deficit tolerance in plants (Cheng et al. 2013). However, ABA treatment suppressed tolerance against water deficit during overexpression of eRF1. It has also been shown that acetic acid treatment induces the expression of eRF1, preceding the expression of ABA-responsive genes (supplementary data in Kim et al. 2017). This relationship between the timing of the expression of eRF1 and its functional suppression suggests that acetic acid-induced drought tolerance precedes ABA-related mechanisms. Moreover, the expression of ABA-responsive genes, such as RD29A, RD20, and so on, was delayed by acetic acid treatment (Kim et al. 2017).

If water stress is sudden and causes an immediate loss of water from the plant body, it seems that the initial phase of physiological response will be skipped. However, the mechanism is highly evolutionarily conserved in both monocots and dicots. Indeed, this treatment can maintain plant survivability under water-limiting conditions, for example, in plants such as cassava, maize, rice, rapeseed, and wheat (Kim et al. 2017; Utsumi et al. 2019; Ogawa et al. 2021). Thus, acetic acid application is useful for maintaining the survivability and productivity of green resources under drought conditions.

## Updates on functional studies on acetic acid for stress resistance in plants

Further evidence of the effectiveness of acetic acid in enhancing drought tolerance and its potential for agricultural applications is accumulating. For example, cassava has been reported as another plant species in which treatment with exogenous acetic acid enhances drought tolerance (Utsumi et al. 2019). Drought-induced overexpression of *PDC1* or *ALDH2B7* results in improved drought tolerance in Arabidopsis (Rasheed et al. 2018). These results support the potential use of exogenous and endogenous acetic acids to protect plants from drought. Meanwhile, it has been pointed out that the membrane permeability of acetic acid is dependent on pH and status of dissociation. Therefore, it is important to understand the mode of action of exogenously supplied acetic acid (Allen and Allen 2020). Conducting further research with this perspective will assist us in refining the application of acetic acid as a biostimulant for drought tolerance, depending on the plant species and soil conditions.

The fate of absorbed acetic acid in rice has been investigated by metabolome analysis using the isotopic tracer technique (Isaji et al. 2018; Ogawa et al. 2021). In the roots of rice seedlings treated with isotope-labelled acetic acid for 4 days, the labels were detected in primary metabolites, such as organic acids (citrate, fumarate, malate, and 2-oxoglutarate) and amino acids (glutamine, glutamate, serine, threonine, and γ-aminobutyric acid), which are downstream of acetyl CoA (Isaji et al. 2018). The major metabolite of exogenously supplied acetic acid in the xylem sap of rice is glutamine (Ogawa et al. 2021). Glutamine in rice roots is predominantly synthesised by ammonium assimilation and then loaded into xylem sap to translocate nitrogen nutrients to aerial parts (Funayama et al. 2013; Kiyomiya et al. 2001), therefore, treatment with acetic acid may affect nitrogen use efficiency. While acetyl-CoA derived from the absorbed acetic acid should also be used for histone acetylation, the allocation of acetic acid between metabolism and protein modification is unknown.

In the current model of acetic acid-mediated drought signalling, both the epigenetic regulatory pathway involving HDA6 and the jasmonate signalling pathway play essential roles (Kim et al. 2017). To uncover the relationship between the two pathways, comprehensive analysis of histone H4 acetylation (H4ac), histone H3 lysine 3 trimethylation (H3K27me3), and transcripts was conducted by employing a *hda6* mutant and jasmonate treatment technique (Vincent et al. 2022). Comparative omics analysis indicated that the two pathways functionally overlap but are independent of the impact on histone modifications, suggesting the complexity of the mechanism underlying abiotic stress response.

Although HDA6 has been characterised as an epigenetic regulator, WRKY transcription factor 1 (WRKY1) has been proposed as another target of HDA6 (Füßl et al. 2018). WRKY1 is a transcription factor implicated in some biological processes, including drought stress response and light and nitrogen signalling (Qiao et al. 2016; Heerah et al. 2019). Transcriptional regulation by WRKY1 may be another pathway involved in acetic acid-mediated drought signalling.

## Perspective on applications using acetic acid to overcome the problems caused by global warming

Generally, high concentrations of exogenous acetic acid are highly toxic to plants. However, it has been shown that this treatment can maintain plant survival under non-watering conditions in cassava, maize, rice, rapeseed, and wheat (Kim et al. 2017; Utsumi et al. 2019; Ogawa et al. 2021). Thus, acetic acid application is useful for maintaining the productivity of green resources under drought conditions.

Much effort has been made to identify a key genetic factor and introduce it to cultivars by crossbreeding, genetic transformation, and genome editing to adjust crop production to the changing climate. Furthermore, the identification of acetic acid-mediated drought signalling in plants has presented an alternative approach to overcome climate change, by harnessing the potential of plants through epigenetic regulation (Kim et al. 2017; Utsumi et al. 2019).

In the first epigenetic approach, acetic acid was employed as an active ingredient of a biostimulant to enhance drought stress resistance. Acetic acid has good social acceptance as it has been used in agriculture and horticulture for a long time. Furthermore, acetic acid is not an expensive reagent; thus, it can be sold at a reasonable price to farmers (Rahman et al. 2019). In terms of versatility, acetic acid-based biostimulants can be effective for a wide range of land plants because the key genes are conserved (Kim et al. 2017; Utsumi et al. 2019). Therefore, breeding, which is a time-consuming process, is not required for the application of acetic acid-based biostimulants. In fact, in addition to the reported plants (i.e. Arabidopsis, rice, maize, wheat, rapeseed, and cassava), we also confirmed the effectiveness of this biostimulant with numerous vegetable species such as tomato, cabbage, broccoli, and lettuce (data not shown).

Recently, ethanol, a metabolite that is quite similar to acetic acid, has emerged as another potential active ingredient to enhance drought and heat-stress resistance in plants (Matsui et al. 2022; Vu et al. 2022). However, the relationship and interaction between acetic acid and ethanol in abiotic stress response is unclear.

Acetic acid-based biostimulants for drought resistance can be useful not only for conventional agriculture but also for other purposes such as desert greening, food production in space, and smart farming. For instance, in smart farming, the biostimulant could be a solution in combination with other techniques, including weather prediction and monitoring soil water content and plant stress levels. To offer a proper solution for this specific situation, further understanding of the mechanisms underlying acetic acid-induced drought resistance is required. The development of new technologies using the epigenetic approach of regulation by histone modifications, histone variants, and DNA methylation is also expected. Coupling solutions between several new materials and genetic approaches will lead to very important and powerful technologies that can help overcome climate change.

## Data Availability

Not applicable.

## Abbreviations

ALDHs: Aldehyde dehydrogenases
ACS: Acetyl-CoA synthetase
ACN1: Acetate non-utilizing 1
JA: Jasmonic acid
PDC1: Pyruvate decarboxylase 1
ALDH2B7: Aldehyde dehydrogenase 2B7
HDA6: Histone deacetylase 6
ABA: Abscisic acid
DREB2A: Dehydration-responsive element binding protein 2
RD29A: Responsive to desiccation 29A
RD29B: Responsive to desiccation 29B
H4ac: Histone H4 acetylations
H3K27me3: Histone H3 Lysine 3 trimethylation
WRKY1: WRKY transcription factor 1
GCN5: General control non-depressible 5
